# Alternative splicing analysis of lignocellulose-degrading enzyme genes and enzyme variants in *Aspergillus niger*

**DOI:** 10.1007/s00253-024-13137-y

**Published:** 2024-04-19

**Authors:** Yifan Xu, Feiyu Dong, Ruoxin Wang, Maria Ajmal, Xinyu Liu, Hui Lin, Hongge Chen

**Affiliations:** College of Life Sciences, Henan Agricultural University, Zhengzhou, 450002 China

**Keywords:** *Aspergillus niger*, RNA-seq, Lignocellulose-degrading enzymes, Alternative splicing

## Abstract

**Abstract:**

Alternative splicing (AS) greatly expands the protein diversity in eukaryotes. Although AS variants have been frequently reported existing in filamentous fungi, it remains unclear whether lignocellulose-degrading enzyme genes in industrially important fungi undergo AS events. In this work, AS events of lignocellulose-degrading enzymes genes in *Aspergillus niger* under two carbon sources (glucose and wheat straw) were investigated by RNA-Seq. The results showed that a total of 23 out of the 56 lignocellulose-degrading enzyme genes had AS events and intron retention was the main type of these AS events. The AS variant enzymes from the annotated endo-β-1,4-xylanase F1 gene (*xynF1*) and the endo-β-1,4-glucanase d gene (*eglD*), noted as XYNF1-AS and EGLD-AS, were characterized compared to their normal splicing products XYNF1 and EGLD, respectively. The AS variant XYNF1-AS displayed xylanase activity whereas XYNF1 did not. As for EGLD-AS and EGLD, neither of them showed annotated endo-β-1,4-glucanase activity. Instead, both showed lytic polysaccharide monooxygenase (LPMO) activity with some differences in catalytic properties. Our work demonstrated that the AS variants in *A. niger* were good sources for discovering novel lignocellulose-degrading enzymes.

**Key points:**

*• AS events were identified in the lignocellulose-degrading enzyme genes of A. niger.*

*• New β-1,4-xylanase and LPMO derived from AS events were characterized.*

**Supplementary Information:**

The online version contains supplementary material available at 10.1007/s00253-024-13137-y.

## Introduction

Lignocellulose, the most abundant agricultural biomass on Earth, has attracted extensive attention due to its huge potential to substitute fossil resources for chemicals and fuel production (Fatma et al. 2018). However, the recalcitrance of lignocellulose to deconstruction hinders the cost-effective bioconversion of lignocellulosic biomass. Therefore, efficient degradation of lignocellulose is considered as a key step for the economically feasible conversion of lignocellulose biomass (Agarwal 2019). Among the various methods for lignocellulose degradation, enzymatic hydrolysis is considered to be the most promising approach for the utilization of biomass, offering advantages such as eco-friendliness, mild reaction conditions, and low energy consumption (Guo et al. 2023).

*Aspergillus niger* is an efficient lignocellulose degrading fungus with ability to produce a wide spectrum of lignocellulose-degrading enzymes, including cellulases, hemicellulases, ligninases, pectinases, and laccases (Pel et al. 2007). Currently, *A. niger* has been tailored to be an industrial strain for production of cellulase, xylanase, and other kind of lignocellulose-degrading enzymes (Cairns et al. 2018). Further mining new lignocellulose-degrading enzymes from *A. niger* is of great significance for lignocellulosic biomass utilization.

Alternative splicing (AS) allows eukaryotic organisms to produce multiple different transcripts from a limited set of genes, generating diverse protein variants and significantly expanding the proteome’s diversity. AS also diversifies the function of enzymes, which has been identified in numerous organisms (Zanini et al. 2022; Yung et al. 2022; Noda et al. 2022). For instance, Tan et al. (2023) identified an AS variant of the tumor suppressor liver kinase B1 (LKB1), noted as mitochondria-localized LKB1, which exhibited distinct tissue distribution, specific expression patterns, and biological functions compared to the normal splicing product LKB1. Boldo et al. (2010) found out an intron-retaining chitinase Chi2 in the filamentous fungi *Metarhizium anisopliae*, which exhibited different biological functions with the normal splicing chitinase. Recently, we identified an intron-retaining β-glucosidase (BGL1B) in *A. niger*, which exhibited a higher optimal temperature and higher thermal stability compared to the normal splicing β-glucosidase (BGL1A) (Zhu et al. 2019). Furthermore, BGL1B had a higher catalytic activity for the hydrolysis of geniposide than that of BGL1A (Zhu et al. 2019).

The identification of the β-glucosidase AS variant BGL1B in *A. niger* (Zhu et al. 2019) inspired us to explore whether other lignocellulose-degrading enzyme genes in *A. niger* also undergo AS events and what particular properties the enzyme variants derived from AS would display. To the best of our knowledge, there have been no other reports on investigation of AS events occurring in *A. niger* genes, particularly in those genes related to lignocellulose-degrading enzymes. In this study, the AS events occurring on lignocellulose-degrading enzyme genes of *A. niger* were investigated, and the AS variant enzymes from annotated endo-β-1,4-xylanase F1 gene (*xynF1*) and endo-β-1,4-glucanase d gene (*eglD*) were characterized. The present work suggests that the AS variants should be good sources for discovering new lignocellulose-degrading enzymes with excellent catalytic properties.

## Materials and methods

### Regents

Xylan from beechwood and Avicel PH-101 were purchased from Sigma-Aldrich (St. Louis, USA). Amplex™ Red Hydrogen Peroxide/Peroxidase Assay Kit was purchased from Thermo Fisher Scientific, Inc. (MA, USA). Q5® High-Fidelity DNA Polymerase and *Dpn*I were from New England Biolabs, Inc. (NE, USA).

### Strain, growth medium, and culture conditions

The *A. niger* CBS513.88 strain in this study was obtained from the Guangdong Microbial Culture Collection Center (Guangzhou, China). The strain was grown at 28 °C on potato dextrose agar (PDA) medium. *A. niger* spores were harvested from the 4-day-old culture on solid medium using sterile deionized water, and the spore suspension (1 × 10^7^ spores mL^−1^) was used as an inoculum. Erlenmeyer flasks (250 mL) containing 50 mL of Mandel’s medium (3 g L^−1^ KH_2_PO_4_, 2 g L^−1^ (NH_4_)_2_SO_4_, 0.5 g L^−1^ MgSO_4_·7H_2_O, 0.5 g L^−1^ CaCl_2_, 7.5 mg L^−1^ FeSO_4_·7H_2_O, 2.5 mg L^−1^ MnSO_4_·H_2_O, 3.6 mg L^−1^ ZnSO_4_·7H_2_O, 0.5 g L^−1^ CoCl_2_·6H_2_O) with 0.1% (w/v) peptone, and 2% (w/v) glucose or 2% (w/v) wheat straw were inoculated with 0.5 mL of the spore suspension. After incubation at 28 °C and 140 rpm for 72 h, the mycelia were harvested by filtration through four-layer cheesecloths.

### Transcriptome analysis

The fungal mycelia of *A. niger* CBS513.88 grown under glucose (G group) or wheat straw (WS group) as a sole carbon source, were collected respectively. The mycelia were washed three times with Mandel’s basal medium, and total RNA from mycelia of glucose groups (G1, G2, G3) and wheat straw groups (WS1, WS2, WS3) was extracted using the RNAiso plus (Takara Bio Inc., Shiga, Japan) reagent following the manufacturer’s instructions. The qualified RNA was subjected to reverse transcription using the 5 × All-In-One RT Master Mix kit (Applied Biological Materials Inc., Richmond, Canada), as per the manufacturer’s protocol, to synthesize the first-strand cDNA. The resulting cDNA was used for subsequent experiments or stored at − 20 °C.

The construction of RNA-seq libraries and sequencing was performed by BGI Genomics. Triplicates of each group were used to construct cDNA libraries. After sequencing on the BGISEQ-500 platform (BGI Genomics Co. Ltd., Shenzhen, China) and quality control procedures, clean data were obtained (NCBI accession number PRJNA1067358). The clean reads were aligned to the reference genome using HISAT2 (Kim et al. 2015), and then, the alignment results were mapped to the reference gene sequences using Bowtie2 (Langmead and Salzberg 2012). Subsequently, RSEM (RNA-Seq by Expectation–Maximization) (Li and Dewey 2011) was employed to calculate gene expression levels for each sample. The genomic sequence and annotation files of *A. niger* CBS513.88 were acquired from the National Center for Biotechnology Information (NCBI), with reference genome version GCF_000002855.3_ASM285v2. DESeq2 (Love et al. 2014), an R package, was utilized to identify differentially expressed genes (DEGs), with a significance threshold set at *P* < 0.05.

### Alternative splicing analysis

The analysis of AS in *A. niger* CBS513.88 was conducted using ABLas. The ABLas analysis algorithm, being frequently employed for AS analysis in the human genome, is based on splice site information (Xiao et al. 2012; Mai et al. 2016). This algorithm was also adapted for the analysis of AS in lower eukaryotic organisms such as fungi (Jin et al. 2017). Briely, TOPHAT2 (Trapnell et al. 2009) was firstly employed to identify splice junctions demarcating annotated exons and introns within the *A. niger* CBS513.88 genome. Then based on read counts at splice junctions, nine distinct AS types were identified, including exon skipping (ES), alternative 5′ splice site (A5SS), alternative 3′ splice site (A3SS), mutually exclusive exons (MXE), mutually exclusive 5′ UTRs (5pMXE), mutually exclusive 3′UTRs (3pMXE), alternative 5′ splice site and exon skipping (A5SS&ES), alternative 3′ splice site and exon skipping (A3SS&ES), cassette exons, and intron retention (IR). In addition, the determination of IR events requires that candidate splicing events meet the following four criteria. (1) The average base depth across candidate introns was at least 20% of the flanking exons’ depth; (2) the cumulative depth of introns exceeded 100; (3) flanking sequences were evident at the 5′ or 3′ splice sites of candidate introns; (4) no other types of AS events were detected.

### Verification of A5SS and IR events in the transcripts of lignocellulose-degrading enzyme genes

Using cDNA obtained by reverse transcription as a template, RT-PCR amplification of *xynF1* (NCBI ID: 4,980,082), *abnC* (NCBI ID: 4,979,546), *cbhC* (NCBI ID: 4,982,491), *bglM* (NCBI ID: 4,984,238), and *eglD* (NCBI ID: 4,988,091) was carried out using gene-specific primers. The PCR products were purified using the DNA Gel Extraction Kit (Sangon Biotech Co., Ltd., Shanghai, China), ligated into the One Step ZTOPO-Blunt/TA vector (Zhuangmeng Technology Co., Ltd., Beijing, China) and transformed into *Escherichia coli* DH5α. The clones were verified by DNA sequencing (Sangon Biotech Co., Ltd., Shanghai, China). Then, intron-spanning primers were designed for PCR validation based on the five lignocellulose-degrading enzyme genes *xynF1*, *abnC*, *cbhC*, *bglM* and *eglD*. The primers are listed in the Supplemental Table S1.

### Expression and purification of normal splicing products and AS variants

The recombinant plasmids were constructed through homologous recombination. First, the pCold-TF vector was linearized by inverse PCR at the site foreign gene would be inserted; second, the foreign gene needed to be expressed was reamplified using primers containing 20-bp homologous arms, corresponding to the 5′ end and 3′ end of the linearized plasmid, respectively; third, the above linearized plasmid and the foreign gene with the homologous arms were recombined using ClonExpress® II One Step Cloning Kit (Vazyme Biotech Co., Ltd., Nanjing, China) and then transformed into *E. coli* DH5α competent cells for selection.

The correct recombinant plasmids were subsequently transformed into *E. coli* BL21 (DE3) for expression. *E. coli* BL21 (DE3) cells harboring the recombinant plasmid were cultured in LB medium supplemented with 50 μg mL^−1^ ampicillin until an absorbency of 600 nm reached 0.6–0.8. Expression of the protein was induced with the addition of isopropyl β-d-1-thiogalactopyranoside (IPTG) to a final concentration of 0.4 mM, and the culture temperature was decreased to 16 °C for 16 h cultivation. The cells were harvested by centrifugation and resuspended in pH 7.0 phosphate buffer. Mini low-temperature ultra high-pressure cell disruptor (NAGO Biotechnology Co., Ltd., Guangzhou, China) was used for cell disruption. After cell disruption, the recombinant proteins in supernatant were purified by Ni^2+^ affinity chromatography (QIAGEN, Hilden, Germany), followed by ultrafiltration method for concentration and desalting. The purified proteins were analyzed by sodium dodecyl sulfate–polyacrylamide gel electrophoresis (SDS-PAGE). The protein concentration was measured by the Bradford method with bovine serum albumin as a standard (Bradford 1976).

### Enzyme assay of the normal splicing product of *xynF1* (XYNF1) and the AS variant of *xynF1* (XYNF1-AS)

Xylanase activities of XYNF1 and XYNF1-AS were measured by the dinitro salicylic acid (DNS) method. The reaction mixture (800 μL) containing 150 mM citrate buffer (pH 5.8), 2.5 mg mL^−1^ birchwood xylan, and 100 μL appropriately diluted enzyme was incubated at 50 °C for 15 min. Then 600 μL of DNS solution was added to terminate the reaction. The reducing sugar released was determined with xylose as a standard. All determinations were performed in triplicate. One unit of xylanase activity (U) was defined as the amount of enzyme that released 1 μmoL of xylose per minute under the above assay conditions.

For kinetic analysis of XYNF1-AS, different concentrations of beechwood xylan (0.2 ~ 15 mg mL^−1^) was used, and the initial reaction rates at each concentration of beechwood xylan were determined under the aforementioned conditions; the reaction was carried out at 50 °C for 5 min. The values of released xylose were fitted on non-linear regression with Michaelis–Menten model (Michaelis and Menten 1913) using GraphPad Prism version 5.0 (GraphPad Software, Boston, USA, www.graphpad.com) to determine *K*_m_ and *k*_cat_.

### Enzyme assay of the normal splicing product of *eglD* (EGLD) and the AS variant of *eglD* (EGLD-AS)

The activity of lytic polysaccharide monooxygenase (LPMO) of EGLD and EGLD-AS was initially measured by the DNS method. The reaction mixture (1000 μL) contained 10 mM ascorbate, 150 mM phosphate buffer (pH 7.0), 2.5 mg mL^−1^ various polysaccharides (Avicel, carboxymethyl cellulose (CMC), filter paper, and xylan), and 100 μL appropriately diluted enzyme. The reaction mixture was incubated at 50 °C for 6 h with shaking at 200 rpm. The reaction was terminated by addition of 600 μL DNS solution, and the reducing sugar released was determined.

LPMO activities of EGLD and EGLD-AS were further measured using the Amplex™ Red Hydrogen Peroxide/Peroxidase Assay Kit, which is based on Amplex Red oxidation by horseradish peroxidase (AR/HRP), and the activity was determined by assaying the H_2_O_2_ produced (Stepnov and Eijsink 2023). The reaction mixture (100 μL) contained 30 μM ascorbate, 100 mM phosphate buffer (pH 7.0), and 20 μL appropriately diluted enzyme. The reaction mixture was incubated at 40 °C for 30 min under light-proof conditions. To stop the reaction, the reaction tubes were placed in an ice water bath immediately after incubation. The absorbance at 560 nm was then measured to determine the H_2_O_2_ concentration. All reactions were performed in triplicate. One unit of LPMO activity (U) was defined as the amount of enzyme that released 1 μmoL of H_2_O_2_ per minute under above assay conditions.

## Results

### Analysis of the AS events of lignocellulose-degrading enzyme genes in *A. niger*

Here, we defined lignocellulose-degrading enzymes as all enzymes that are relevant to lignocellulose degradation (Mach-Aigner et al. 2012), including β-glucosidase (EC3.2.1.21), cellobiohydrolase (EC3.2.1.91), endo-β-1,4-glucanase (EC3.2.1.4), endo-β-1,4-xylanase (EC3.2.1.8), β-xylosidase (EC3.2.1.37), α-l-arabinofuranosidase (EC3.2.1.55), and acetyl xylan esterase (EC3.1.1.72), auxiliary activities (AA) families, and carbohydrate-binding modules (CBM) families. Based on the genome of *A. niger* CBS513.88, 56 genes were considered as lignocellulose-degrading enzyme genes in *A. niger* (Supplemental Table S2).

To investigate the AS events of lignocellulose-degrading enzyme genes in *A. niger* CBS513.88, two culture conditions were performed: growth medium with glucose as a sole carbon source (named G group) and enzyme-producing medium with wheat straw as a sole carbon source (named WS group). RNA-seq analysis of mycelia from two groups showed that 33 out of the 56 lignocellulose-degrading enzyme genes in the WS group had higher expressions than that in the G group, which is consistent with the previous study (Pullan et al. 2014). It is noticeable that about 2% of sequence reads were derived from intron regions in both G and WS groups, which suggested that some annotated introns were retained in mRNA and AS events might exist.

Based on the RNA sequencing, altogether 23 genes of the 56 lignocellulose-degrading enzyme genes were found to have AS events. Specifically, 13 genes in G group and 14 genes in WS group showed AS events (Table 1). Regarding AS types occurring in two groups, four AS types were detected including exon skipping (ES), alternative 5′ splice site (A5SS), alternative 3′ splice site (A3SS), and intron retention (IR). However, IR type was the predominant type, accounting for 95.6% of all AS events occurring in two groups.

**Table 1:** AS events of lignocellulose-degrading enzyme genes in *A. niger* CBS513.88 under two carbon source conditions

NCBI ID	Gene name	AS types	
		WS group	G group
4980363	*bgl1B*	-	IR
4980522	*bglJ*	IR	-
4982032	*bglG*	IR	-
4983142	*bglD*	IR	-
4984238	*bglM*	IR	IR
4987033	*bglM*	-	IR
4989339	*bgl*	-	IR
4982202	*cbhB*	-	IR
4982491	*cbhC*	IR	-
4985573	*cbhC*	IR	-
4989072	*eglA*	IR	-
4977958	*xynA*	IR	-
4980082	*xynF1*	A5SS&A3SS	-
4978177	*abfA*	IR	IR
4982486	*abfC*	-	IR
4979546	*abnC*	IR	-
4983631	-	-	IR
4991259	*gun4*	IR	IR
4980991	*abr2*	-	IR
4978139	-	IR	-
4988091	*eglD*	IR	IR
4988766	-	IR	ES/IR
4987123	*gun4*	IR	IR

“-” indicates that none of the AS type was detected in all 3 replicate samples. Genes marked with AS types mean that the AS types occurred in at least 1 of 3 replicate samples. When different AS types were detected in 3 replicate samples, separate them with “/”

### Identification of AS variants of lignocellulose-degrading enzyme genes

**Fig. 1:**
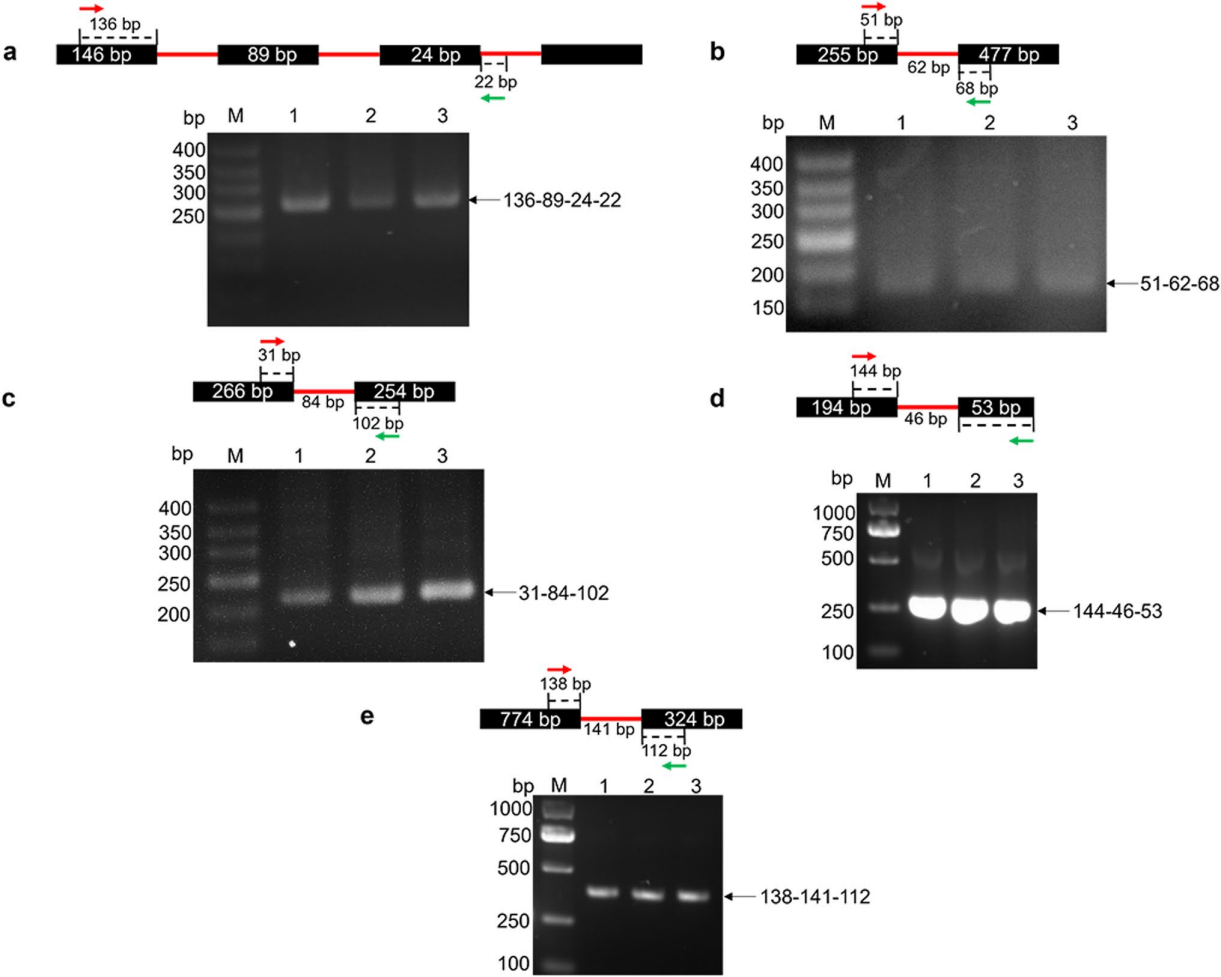
Intron-specific amplification of the selected lignocellulose-degrading enzyme genes. M: 50 bp DNA ladder; 1, 2, 3: three samples in WS group; **a** The validation of the A5SS&A3SS event of the *xynF1* gene. **b** The validation of the IR event (retention of the 1st intron) in the *abnC* gene. **c** The validation of the IR event (retention of the 1st intron) in the *cbhC* gene. **d** The validation of the IR event (retention of the 2nd intron) in the *bglM* gene. **e** Validation of the IR event (retention of the 1st intron) in the *eglD* gene. In the schematic diagram, black squares represent exons, red thin lines represent introns, red and green arrows represent annealing positions of primers, and black arrows point to the amplification products, the sizes of which are the sum of all fragment lengths marked besides the arrows

Endo-β-1,4-glucanase, cellobiohydrolase, β-glucosidase, endo-β-1,4-xylanase, and arabinofuranosidase are essentially important hydrolases for the degradation of lignocellulose. All these enzymes’ genes were found to have AS events in WS group. Here, one gene from each of the five hydrolases was picked up for further identifying its AS variants. The genes selected for this purpose included endo-β-1,4-xylanase gene *xynF1* (NCBI ID: 4980082), arabinofuranosidase gene *abnC* (NCBI ID: 4979546), cellobiohydrolase gene *cbhC* (NCBI ID: 4982491), β-glucosidase gene *bglM* (NCBI ID: 4984238), and endo-β-1,4-glucanase gene *eglD* (NCBI ID: 4988091). The reverse transcription PCR of these five genes showed that the sizes of the products were consistent with the theoretical mRNA sizes of these five genes (Supplemental Fig. S1). To determine the presence of normal and alternative splicing transcripts, the RT-PCR products were cloned into ZTOPO-Blunt-T and transferred into *E. coli* DH5α. Ten transformants for each gene were picked up for sequencing, and the results showed that both normal and alternative splicing transcripts were detected in all five genes, but the number of normal and alternative splicing transcripts was different (Supplemental Table S3). For those transformants identified to carry alternative splicing transcripts, the intron-spanning primers of these five genes were designed to amplify the regions involved in AS events. The results indicated that the intronic sequences of all five genes were present, which further confirmed their AS events (Fig. 1).

**Fig. 2:**
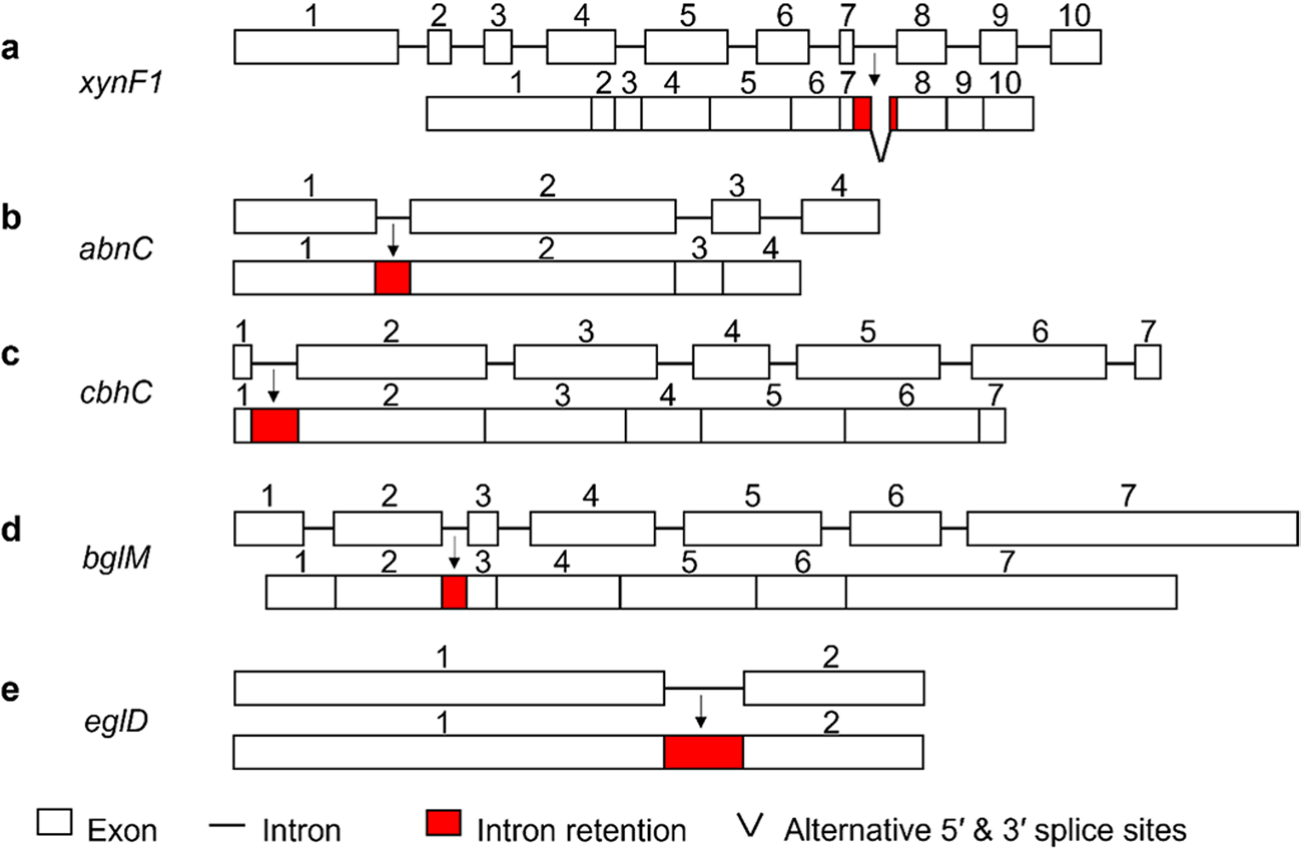
Schematic of AS types of the selected lignocellulose-degrading enzyme genes. Upper: the genomic DNA of each gene; below: AS variant

The sequencing results of the intron-spanning PCR products also proved the AS types that occurred on the five genes. For AS event in the *xynF1* gene, the first 22 bp of the 5′ end of the 7th intron and the last 2 bp of the 3′ end of the same intron were retained in transcript, resulting in an A5SS&A3SS AS event (Fig. 2a). All the other four genes displayed IR type of AS events with the *abnC* gene retaining the 1st intron (62 bp) (Fig. 2b), the *cbhC* gene retaining the 1st intron (84 bp) (Fig. 2c), the *bglM* gene retaining the 2nd intron (46 bp) (Fig. 2d), and the *eglD* gene retaining the 1st intron (141 bp) (Fig. 2e).

The putative translation of the AS variants of the five genes (Table 2) showed that the AS variants of the genes *abnC*, *cbhC*, and *bglM* led to premature translation termination at the 102nd, 15th, and 132nd amino acid positions, respectively, resulting in non-functional proteins, while the AS variants of the *xynF1* and *eglD* genes had 8 and 47 additional amino acids compared with their normal splicing products, respectively (Supplemental Fig. S2 and Supplemental Fig. S3). Therefore, the AS variant of *xynF1* (named XYNF1-AS) and the AS variant of *eglD* (named EGLD-AS) were selected to see whether they exhibited different enzymatic functions compared to the normal splicing products of *xynF1* and *eglD*, named XYNF1 and EGLD, respectively.

**Table 2:** Putative translational products of AS variants of the selected lignocellulose-degrading enzyme genes

Candidate gene	AS types	Putative translation product of normal splicing transcript	Putative translation product of AS transcript
*xynF1*	A5SS	319 aa	327 aa
*abnC*	IR	318 aa	Termination at the 102nd aa
*cbhC*	IR	435 aa	Termination at the 15th aa
*bglM*	IR	765 aa	Termination at the 132nd aa
*eglD*	IR	365 aa	412 aa

### Enzymatic properties of XYNF1 and the AS variant XYNF1-AS

XYNF1 was annotated as an endo-β-1,4-xylanase with a low sequence identity (20.2–36.5%) to GH10 family xylanases (Supplemental Fig. S4). Yet XYNF1 has not been characterized. Both XYNF1 and XYNF1-AS were heterologously expressed in *E. coli* BL21 and purified by Ni–NTA affinity chromatography (Supplemental Fig. S5a). Xylanase activity analysis showed that XYNF1-AS displayed high xylanase activity (3305.7 U mg^−1^), unexpectedly, XYNF1 did not display any activity toward xylan. Therefore, only XYNF1-AS was further characterized.

**Fig. 3:**
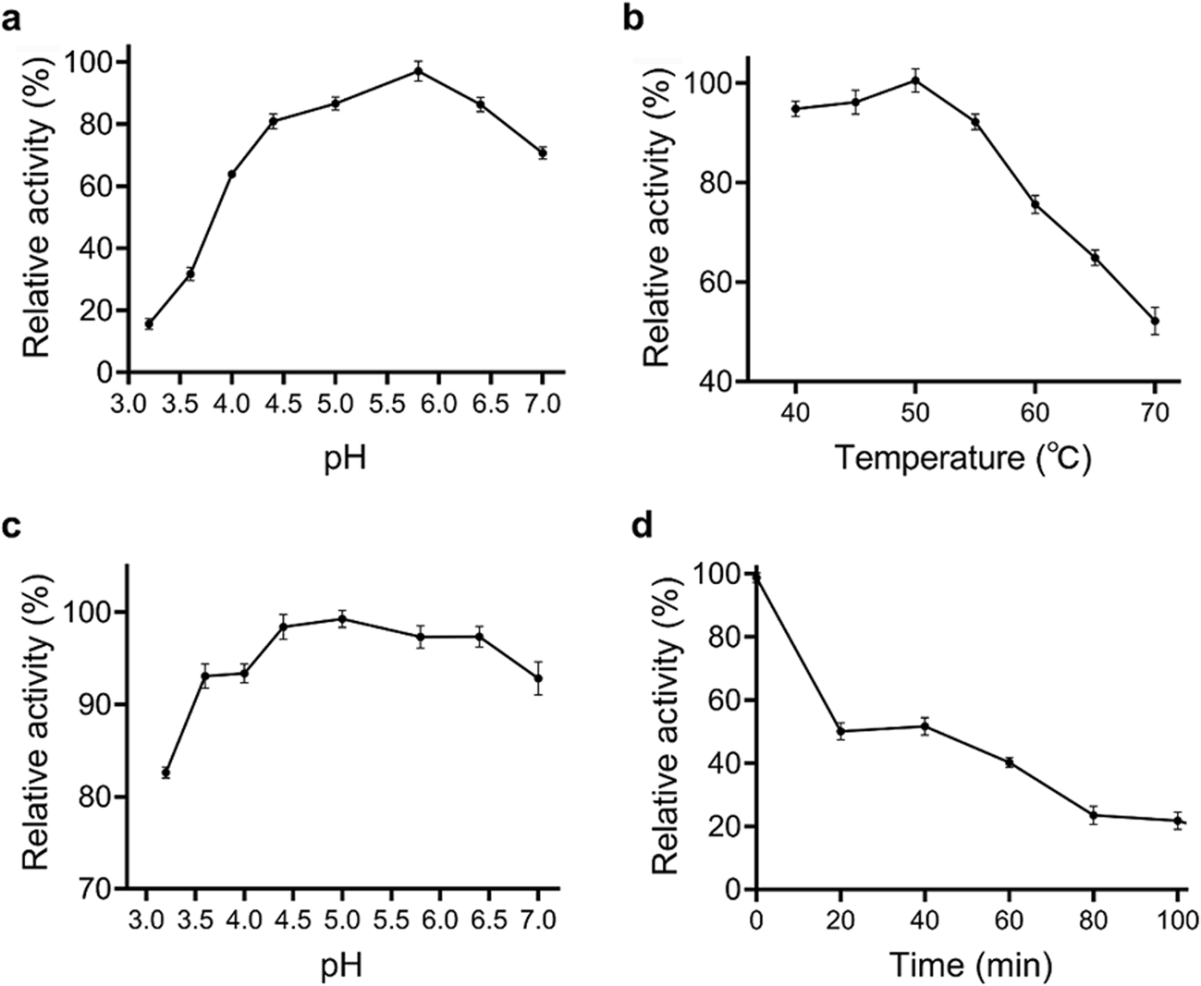
Effects of pH and temperature on activity and stability of XYNF1-AS. **a** Optimal pH of XYNF1-AS. **b** Optimal temperature of XYNF1-AS. **c** pH Stability of XYNF1-AS at pH 3.2–7.0 citrate buffer. **d** Thermal stability of XYNF1-AS at 50 °C

XYNF1-AS had the highest catalytic activity at pH 5.8 and 50 °C (Fig. 3), similar with that of most GH10 xylanases which showed high catalytic activity in acidic condition (Wang et al. 2019). XYNF1-AS showed a high pH stability (Fig. 3c), however, it had a low thermal stability, with 50% residual activity after 40 min of incubation at 50 °C and less than 25% residual activity after 80 min of incubation at 50 °C (Fig. 3d).

The kinetic parameters of XYNF1-AS were then determined with different concentrations of beechwood xylan at pH 5.8 and 50 °C. The results showed that XYNF1-AS had *K*_m_ of 7.41 mg mL^−1^ and *k*_cat_ of 19.51 min^−1^. XYNF1-AS did not display superior catalytic properties compared to known GH10 xylanases (Glekas et al. 2022; Lafond et al. 2011; You et al. 2019).

### Enzymatic properties of EGLD and the AS variant EGLD-AS

**Fig. 4:**
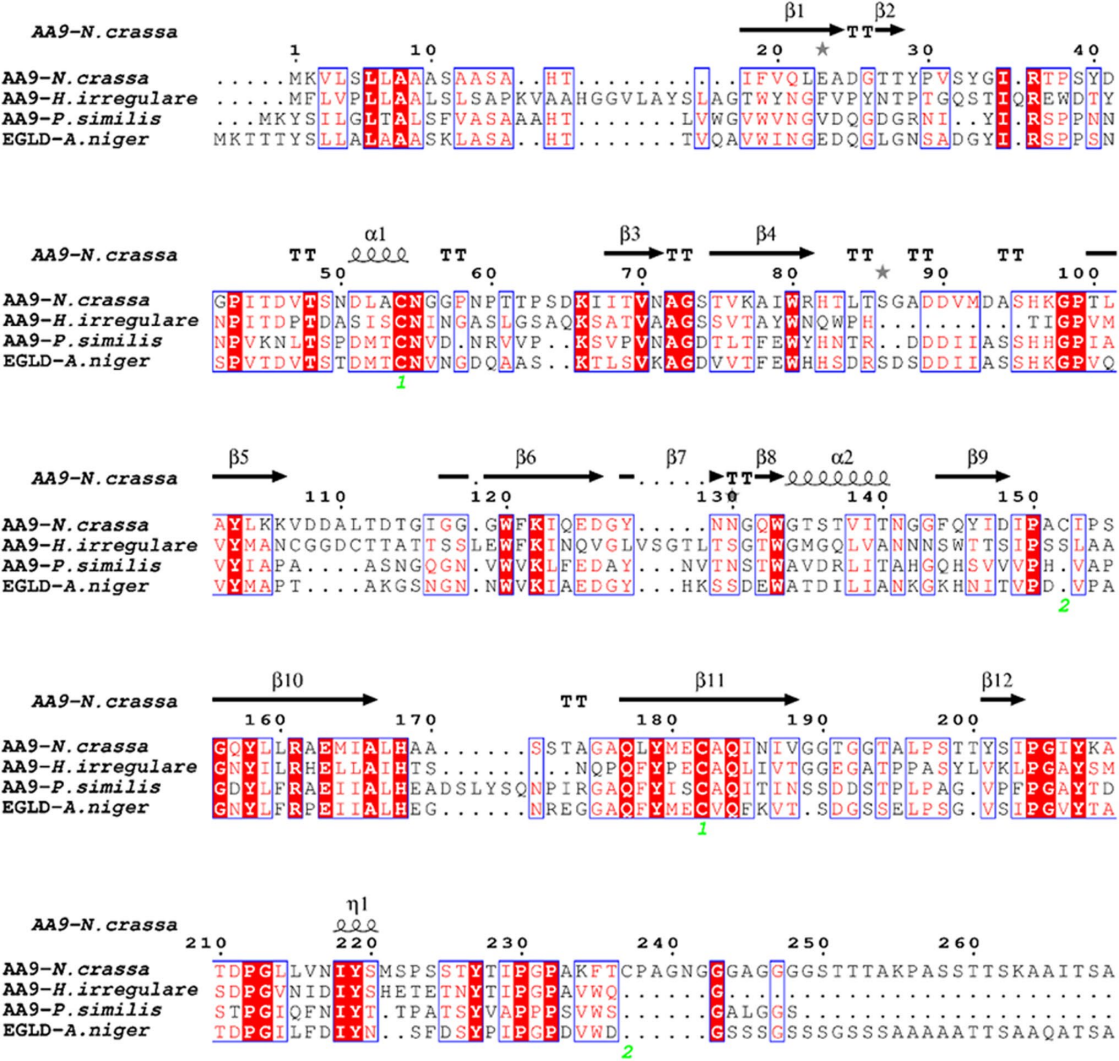
Amino acid sequence alignment of EGLD with AA9 family proteins. The alignment includes AA9 proteins from *Neurospora crassa* (NCBI ID: EAA30263.1), *Heterobasidion irregulare* (NCBI ID: ETW87087.1), *Panus similis* (NCBI ID: ALN96977.1), and EGLD from *A. niger* CBS513.88 in this study (NCBI ID: CAK42466.1). The alignment is numbered according to AA9 protein from *N. crassa*. Fully conserved residues appear in white on a red background, whereas less-conserved residues appear as red letters

EGLD is annotated as a putative endo-β-1,4-glucanase with a carbohydrate-binding module 33 (CBM33). Both EGLD and EGLD-AS were heterologously expressed in *E. coli* BL21 and purified (Supplemental Fig. S5b). Here, the catalytic activities of EGLD and EGLD-AS were firstly assayed using carboxymethyl cellulose (CMC), Avicel, and filter paper as substrates. However, both EGLD and EGLD-AS could not hydrolyze these substrates, indicating that they did not function as the annotated endo-β-1,4-glucanase. The further sequence alignment of EGLD showed that it contained the conserved domain of AA9 family, a typical core β-sandwich structure (Fig. 4). Basically, AA9 family consists mainly of lytic polysaccharide monooxygenases (LPMOs), which can oxidize and cleave glycosidic bonds in cellulose and other polysaccharides in the presence of an external electron donor (Du et al. 2018).

**Fig. 5:**
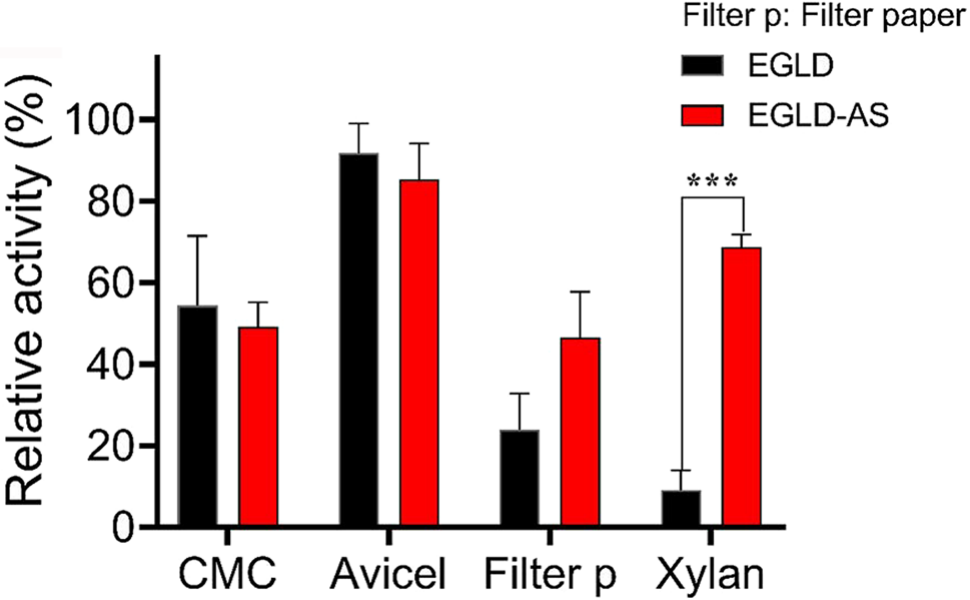
LPMO activities of EGLD and EGLD-AS in terms of cleaving polysaccharides. Note: the reaction system contained a final concentration of 10 mM ascorbate as the electron donor

Therefore, the catalytic activity of EGLD and EGLD-AS for cleaving CMC, Avicel, filter paper, or xylan was assayed in the presence of ascorbic acid as an electron donor (Fig. 5). The results showed that both EGLD and EGLD-AS were able to hydrolyze those polysaccharides in the presence of ascorbic acid, indicating that EGLD and EGLD-AS possessed LPMO activity. To further confirm their LPMO catalytic activity, the oxidative activity of EGLD and EGLD-AS was determined based on Amplex Red oxidation by horseradish peroxidase (AR/HRP). The results proved that EGLD and EGLD-AS displayed the oxidative activities that LPMOs featured.

Both EGLD and EGLD-AS exhibited cleavage polysaccharide activities toward all four tested substrates, indicating that they could catalyze the oxidative cleavage of both β-d-1,4-glucosidic and β-d-1,4-xylosidic bonds in polysaccharide. EGLD and EGLD-AS had similar catalytic activity toward CMC, Avicel, and filter paper, while EGLD-AS exhibited sevenfold higher catalytic activity than that of EGLD when acting on xylan (Fig. 5). However, when oxidative activity based on Amplex Red oxidation was concerned, EGLD showed a higher activity (0.09 U mg^−1^) than that of EGLD-AS (0.05 U mg^−1^).

**Fig. 6:**
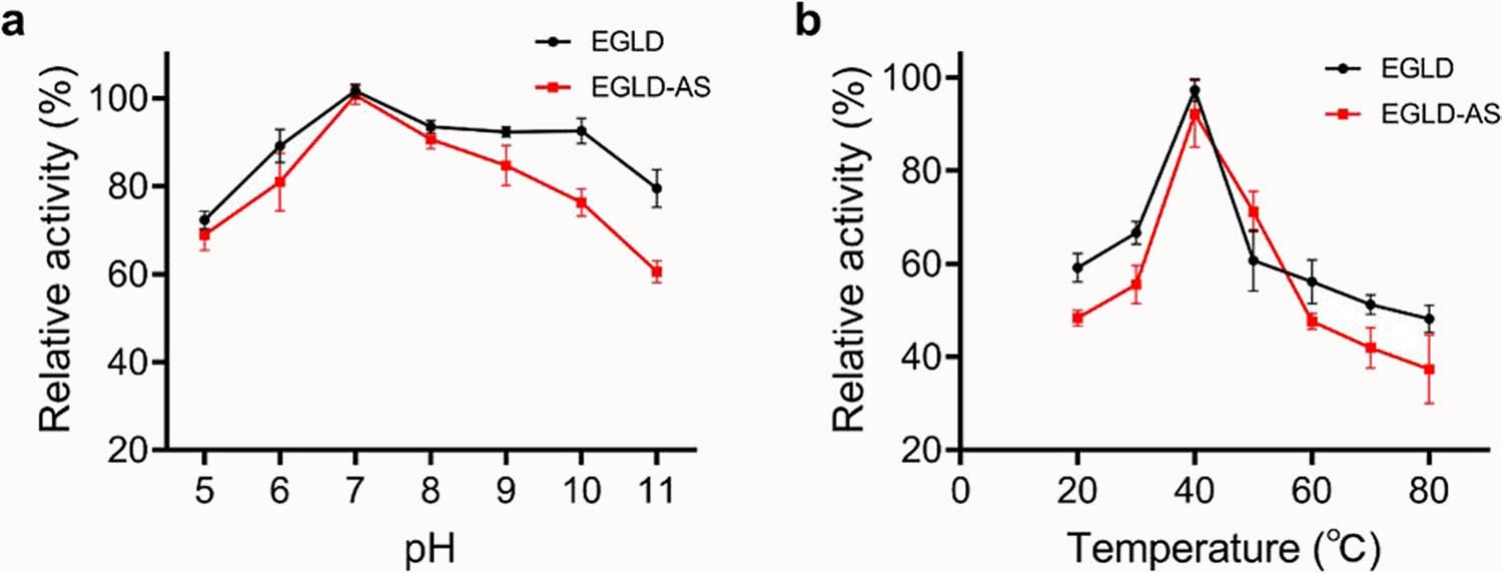
Effects of pH and temperature on LPMO activity of EGLD and EGLD-AS using the Amplex™ Red Hydrogen Peroxide/Peroxidase Assay. **a** Optimal temperature of EGLD and EGLD-AS. **b** Optimal pH of EGLD and EGLD-AS

The effects of pH and temperature on the catalytic activity of both EGLD and EGLD-AS were investigated. The two enzymes exhibited the same optimal pH (pH 7.0) and optimal temperature (40 °C) with very similar pH-dependence profiles and temperature-dependence profiles (Fig. 6).

## Discussion

Alternative splicing represents one of the most prevalent post-transcriptional regulatory mechanisms in eukaryotes. In this study, the AS events of lignocellulose-degrading enzyme genes in *A. niger* grown on glucose medium (G group) and wheat straw medium (WS group) were thoroughly investigated. The genes with AS events under the two growth conditions were different. For example, the AS events of *xynF1*, *abnC*, and *cbhC* were detected in WS group, but they were not detected in G group, which suggested that the growth conditions might affect the occurrence of AS events. Several other studies also observed that AS events were related to growth conditions. For instance, abiotic stress directly impacted the occurrence of AS events in cassava plants, and low temperatures (4 °C), and drought could lead to changes of the abundance of the AS transcripts (Li et al. 2020). It is well known that lignocellulose-degrading enzyme genes in *A. niger* display very different expression profiles when grown on glucose medium and on lignocellulosic medium (Pullan et al. 2014). Our work also demonstrated that lignocellulose-degrading enzyme genes had higher expression level in WS group than in G group. Here, we observed that the occurrence of AS events on lignocellulose-degrading enzyme genes was different between G group and WS group. We then presumed that AS might be involved in the regulation of the expression of lignocellulose-degrading enzyme genes. More work should be done to reveal the regulatory mechanism of AS on the synthesis of lignocellulose-degrading enzymes in *A. niger*.

Since the discovery of AS in 1980 (Choi et al. 1980), numerous studies have shown that AS can produce protein variants with different biological functions. AS variants have frequently been reported to differ from the normal splicing products in terms of their catalytic capability, subcellular localization, or protein–protein interactions (Lan et al. 2022; Acuña et al. 2023). In present work, by analyzing AS events in *A. niger* we identified a new endo-β-1,4-xylanase XYNF1-AS which was an AS variant of *xynF1* gene, and two LPMO enzymes, the normal splicing product EGLD and the AS variant EGLD-AS. It is worth noting that EGLD-AS characterized here was previously reported by Du et al. (2018) as a normal splicing product, the function of which was also determined as a LPMO. AS may enable *A. niger* to produce lignocellulose-degrading enzymes with different properties, which could provide *A. niger* with a multitude of lignocellulose-degrading enzymes to efficiently target different components of lignocellulose. Given that a high ratio of lignocellulose-degrading enzyme genes in *A. niger* (23 out of 56) has been detected to have AS events, the functional AS variants may provide useful sources for discovery of novel lignocellulose-degrading enzymes. Yet, it is noteworthy that not all AS events are biologically meaningful. Some AS transcripts might not be as stable as normally spliced transcripts, resulting in mRNA degradation before protein products formed. Other AS transcripts might form premature proteins due to introduction of stop codons as was seen with the AS events of the genes *abnC*, *cbhC*, and *bglM* in this work.

Currently, the AS variants of *xynF1* and *eglD* have only been characterized in vitro; it is not clear whether these AS variant proteins exist in *A. niger* cells and whether EGLD and EGLD-AS perform different functions if they existed. Answering these questions may provide more understandings about how lignocellulolytic fungi utilize complex lignocellulosic substrates.

In conclusion, we proved the occurrence of AS events on lignocellulose-degrading enzyme genes in *A. niger*. New endo-β-1,4-xylanase and LPMO derived from AS events have been characterized. We proposed that AS is a strategy for *A. niger* to increase the functional diversity of its lignocellulose-degrading enzyme repertoire and AS variants are good sources for discovering novel lignocellulose-degrading enzymes.

## Supplementary Information

Below is the link to the electronic supplementary material.

Supplementary file1 (PDF 716 KB)

## Data Availability

All data generated or analyzed during this study are included in this published article (and its supplementary information files). The RNA-seq raw reads were submitted to the NCBI website’s Sequence Read Archive database under accession number PRJNA1067358.
