# Supplementary material for: Alternative splicing analysis of lignocellulose-degrading enzyme genes and enzyme variants in Aspergillus niger
Source: Appl Microbiol Biotechnol. 2024 Apr 19;108:302. doi: 10.1007/s00253-024-13137-y (PMC11031446; doi:10.1007/s00253-024-13137-y)
Supplement: Supplementary file 1 [file 253_2024_13137_MOESM1_ESM.pdf]

Alternative splicing analysis of lignocellulose-degrading enzyme genes and enzyme variants in *Aspergillus niger*

Yifan Xu, Feiyu Dong, Ruoxin Wang, Maria Ajmal, Xinyu Liu, Hui Lin* & Hongge Chen*

College of Life Sciences, Henan Agricultural University, Zhengzhou 450002, China

*Corresponding authors

Email: huilin@henau.edu.cn

Email: honggeyz@henau.edu.cn

ORCID ID: 0000-0002-7519-7341

Table S1 RT-PCR primers and intron-specific amplification primers for lignocellulose-degrading enzyme genes in *Aspergillus niger*.

Gene name	NCBI ID	RT-PCR primer sequence (5'-3')	
		Forward	Reverse
xynF1	4980082	ATGGTTCAGATCAAGGTAGCTGCA	CTAGAGAGCATTTGCGATAGCAGTG
abnC	4979546	ATGCTTTCGTTTGTCTTGCTTCT	TCACGCCACAACAGGCCA
cbhC	4982491	ATGTTTAGCCGGATTTACTTTGTCACT	TCAAAGGGAAGGATTGGCGT
bglM	4984238	ATGCACAGCATTAGTGCGCT	TTAGATAGAAAATTCCCCCGTCA
eglD	4988091	ATGAAGACTACCACCTACAGTT	TTACTGAGACGCAACGCA
		Intron spanning primer sequence (5'-3')	
xynF1	4980082	ATGAAATCTTCAACGAAGACGG	CCAGAAATTCCAGCACCTCCAC
abnC	4979546	GCACTGCCTAACGGATCCATC	CGGCTGCCCAGGGTAGAGCTA
cbhC	4982491	ATGTTTAGCCGGATTTACTTTGTCACT	GAGAAACGAGGGGCCAGATGG
		GCTGTCTAGTGCTGGGTTTGA	CCTTGACATTGCACCAGTCAC
bglM	4984238	GCCTTTGTCGCCCAGTTGA	CTGGCACCGACGTGAATACC
eglD	4988091	GACCCCGGTATCCTCTTCGAT	CAACCTTGGACTTGGACTTGG

Table S2 Lignocellulose-degrading enzyme genes in the *A. niger* CBS513.88 genome.

Number	NCBI ID	Gene name	Gene annotation	Family	DEG or no DEG
1	4977095	*xg74*	Xyloglucanase	GH74	yes
2	4977345	*cbhB*	1,4-β-D-glucan cellobiohydrolase B	GH7	yes
3	4982202	*cbhB*	1,4-β-D-glucan cellobiohydrolase B	GH7	yes
4	4987438	*aguA*	α-Glucuronidase A	GH67	yes
5	4982491	*cbhC*	1,4-β-D-glucan cellobiohydrolase C	GH6	yes
6	4985573	*cbhC*	1,4-β-D-glucan cellobiohydrolase C	GH6	yes
7	4987831	*abfB*	α-N-arabinofuranosidase B	GH54	yes
8	4978177	*abfA*	α-N-arabinofuranosidase A	GH51	no
9	4982486	*abfC*	α-N-arabinofuranosidase C	GH51	yes
10	4983621	*abfA*	α-N-arabinofuranosidase A	GH51	no

Number	NCBI ID	Gene name	Gene annotation	Family	DEG or no DEG
11	4977614	*eglA*	Endo-β-1,4-glucanase A	GH5	yes
12	4982163	*eglB*	Endo-β-1,4-glucanase B	GH5	yes
13	4989072	*eglA*	Endoglucanase A	GH5	no
14	4979546	*abnC*	α-L-arabinosidase C	GH43	yes
15	4980522	*bglJ*	β-Glucosidase J	GH3	yes
16	4981203	*bglB*	β-Glucosidase B	GH3	no
17	4982032	*bglG*	β-Glucosidase G	GH3	yes
18	4982244	*bglD*	β-Glucosidase D	GH3	yes
19	4983142	*bglD*	β-Glucosidase D	GH3	no
20	4984238	*bglM*	β-Glucosidase M	GH3	yes
21	4987033	*bglM*	β-Glucosidase M	GH3	no
22	4988081	*bglG*	β-Glucosidase G	GH3	yes
23	4989339	*bgl*	β-Glucosidase	GH3	yes
24	4989375	*bglJ*	β-Glucosidase J	GH3	no
25	4989921	*bglA*	β-Glucosidase A	GH3	yes
26	4977682	*xlnD*	Exo-1,4-β-xylosidase D	GH3	yes
27	4977958	*xynA*	Endo-1,4-β-xylanase A	GH11	yes
28	4978152	*xynA*	Endo-1,4-β-xylanase A	GH11	yes
29	4987596	*xyn5*	Endo-1,4-β-xylanase 5	GH11	yes
30	4988056	*xynB*	Endo-1,4-β-xylanase B	GH11	yes
31	4980082	*xynF1*	Endo-1,4-β-xylanase F1	GH10	yes
32	4980363	*bgl1B*	β-Glucosidase G	GH1	yes
33	4983612	-	Acetyl xylan esterase	CE5	no
34	4983631	-	Acetyl xylan esterase	CE5	yes
35	4985851	*axeA*	Acetyl xylan esterase A	CE1	yes
36	4980408	-	Hypothetical protein	CBM63	yes
37	4978139	-	Hypothetical protein	CBM50	no
38	4988766	-	Hypothetical protein	CBM48	yes
39	4979015	-	Hypothetical protein	CBM21	yes
40	4988091	*eglD*	Probable endo-β-1,4-glucanase D	CBM1/AA9	yes
41	4987123	*gun4*	Endoglucanase-4	CBM1/AA9	yes
42	4988058	-	Hypothetical protein	CBM1	yes
43	4982839	*gun4*	Endoglucanase-4	AA9	no

Number	NCBI ID	Gene name	Gene annotation	Family	DEG or no DEG
44	4985809	*gun4*	Endoglucanase-4	AA9	yes
45	4991259	*gun4*	Endoglucanase-4	AA9	no
46	4979417	*cdh*	Cellobiose dehydrogenase	AA2	yes
47	4990292	*cdh*	Cellobiose dehydrogenase	AA2	yes
48	4978017	*abr2*	Laccase abr2	AA1	yes
49	4977636	*abr2*	Laccase abr2	AA1	yes
50	4980364	*abr2*	Laccase abr2	AA1	no
51	4980991	*abr2*	Laccase abr2	AA1	yes
52	4991441	*abr2*	Laccase abr2	AA1	no
53	4985931	*lac1*	Laccase ARB	AA1	yes
54	4988595	*lac1*	Laccase ARB	AA1	yes
55	4980971	*lac2*	Laccase-2	AA1	no
56	4984589	*lac2*	Laccase-2	AA1	no

Note: DEG stands for differentially expressed genes.

Table S3 DNA sequencing verification of AS events in five selected lignocellulose-degrading enzyme genes

Gene name	Number of normal splicing transcripts in 10 transformants	Number of AS transcripts in 10 transformants
xynF1	4	6
abnC	8	2
cbhC	7	3
bglM	6	4
eglD	2	8

Note: RT-PCR products of each gene were randomly cloned and 10 transformants were selected for DNA sequencing.

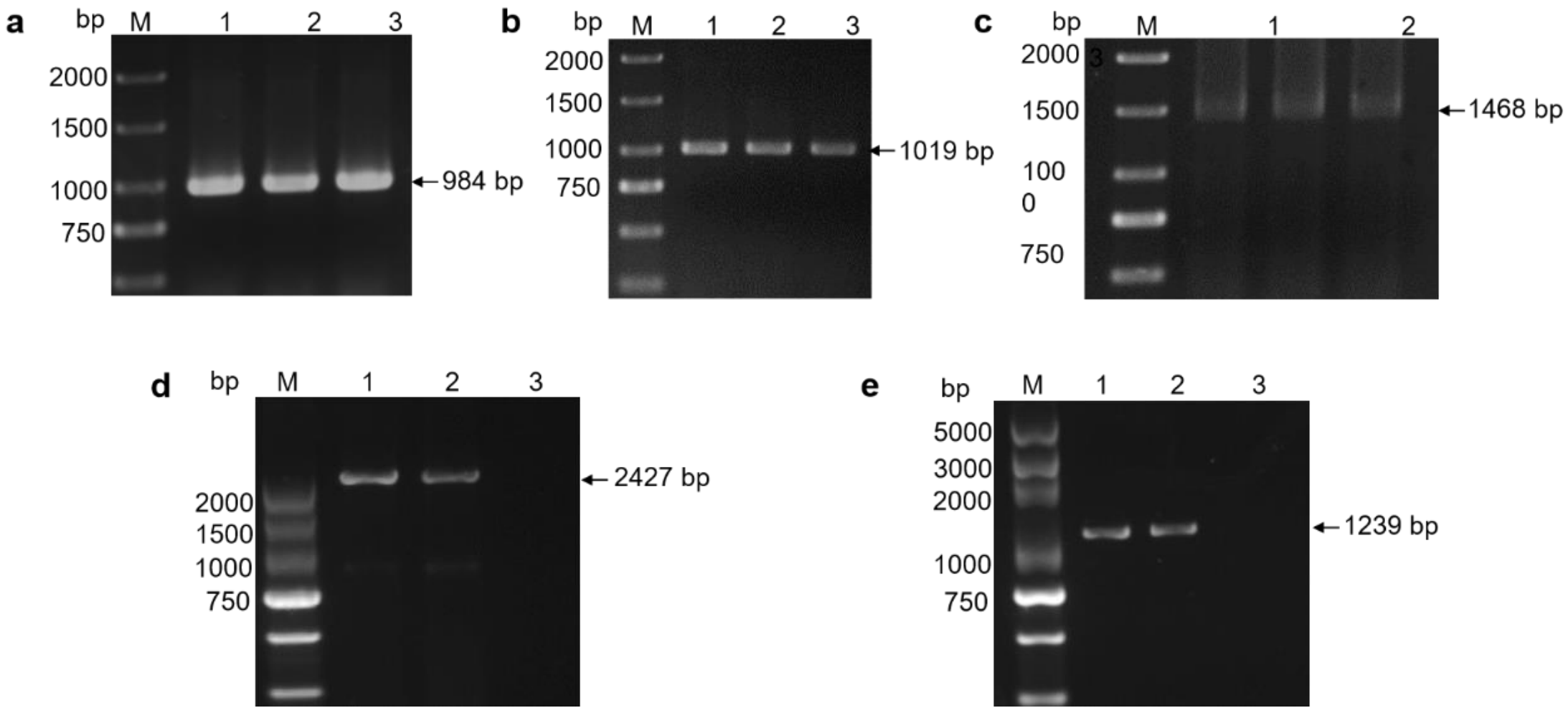

Fig. S1 RT-PCR amplification of the selected lignocellulose-degrading enzyme genes. The theoretical size of the amplified genes were marked. M: DL2000 Marker (Zhuangmeng Technology Co., Ltd., Beijing, China); 1, 2, 3: three samples in the WS group. **a** The RT-PCR electrophoresis image of the *xynF1* gene. **b** The RT-PCR electrophoresis image of the *abnC* gene. **c** The RT-PCR electrophoresis image of the *cbhC* gene. **d** The RT-PCR electrophoresis image of the *bglM* gene. **e** The RT-PCR electrophoresis image of the *eglD* gene.

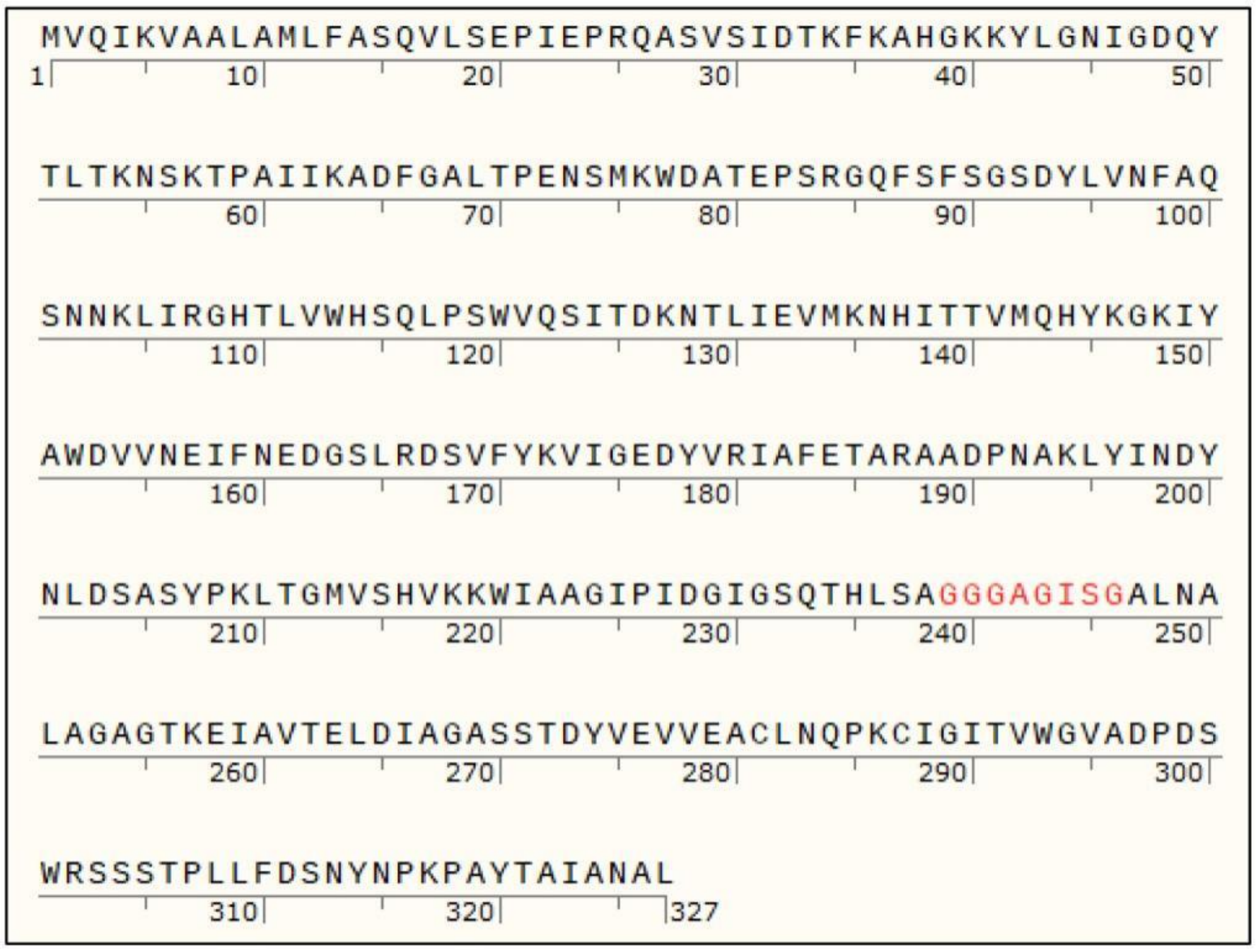

Fig. S2 Amino acid sequence of the AS variant XYNF1-AS. Amino acid changes caused by AS events are colored in red.

```
MKTTTYSLLALAAASKLASAHTTVQAVWINGEDQGLGNSADGYIRSPPSN
1        10        20        30        40        50

SPVTDVTSTDMTCNVNGDQAASKTLSVKAGDVVTFEWHHSDRSDSDDIIA
         60        70        80        90       100

SSHKGPVQVYMAPTAKGSNGNNWVKIAEDGYHKSSDEWATDILIANKGKH
        110       120       130       140       150

NITVPDVPAGNYLFRPEIIALHEGNREGGAQFYMECVQFKVTSDGSSELP
        160       170       180       190       200

SGVSIPGVYTATDPGILFDIYNSFDSYPIPGPDVWDGSSSGSSSGSSSAA
        210       220       230       240       250

AAATTSAAVAATTPATQAAVEVSSSAAAVVESTSSAAAATTEAAAPVVSS
        260       270       280       290       300

AAPVQQATSAVTSQAQAPTTFATSSKSSKTACKNKTKSKSKVAASSTEAV
        310       320       330       340       350

VAPAPTSSVVPAVSASASASAGGVAKMYERCGGINHTGPTTCESGSVCKK
        360       370       380       390       400

WNPYYYQCVASQLE
        410   414
```

Fig. S3 Amino acid sequence of the AS variant EGLD-AS. Amino acid changes caused by AS events are colored in red.

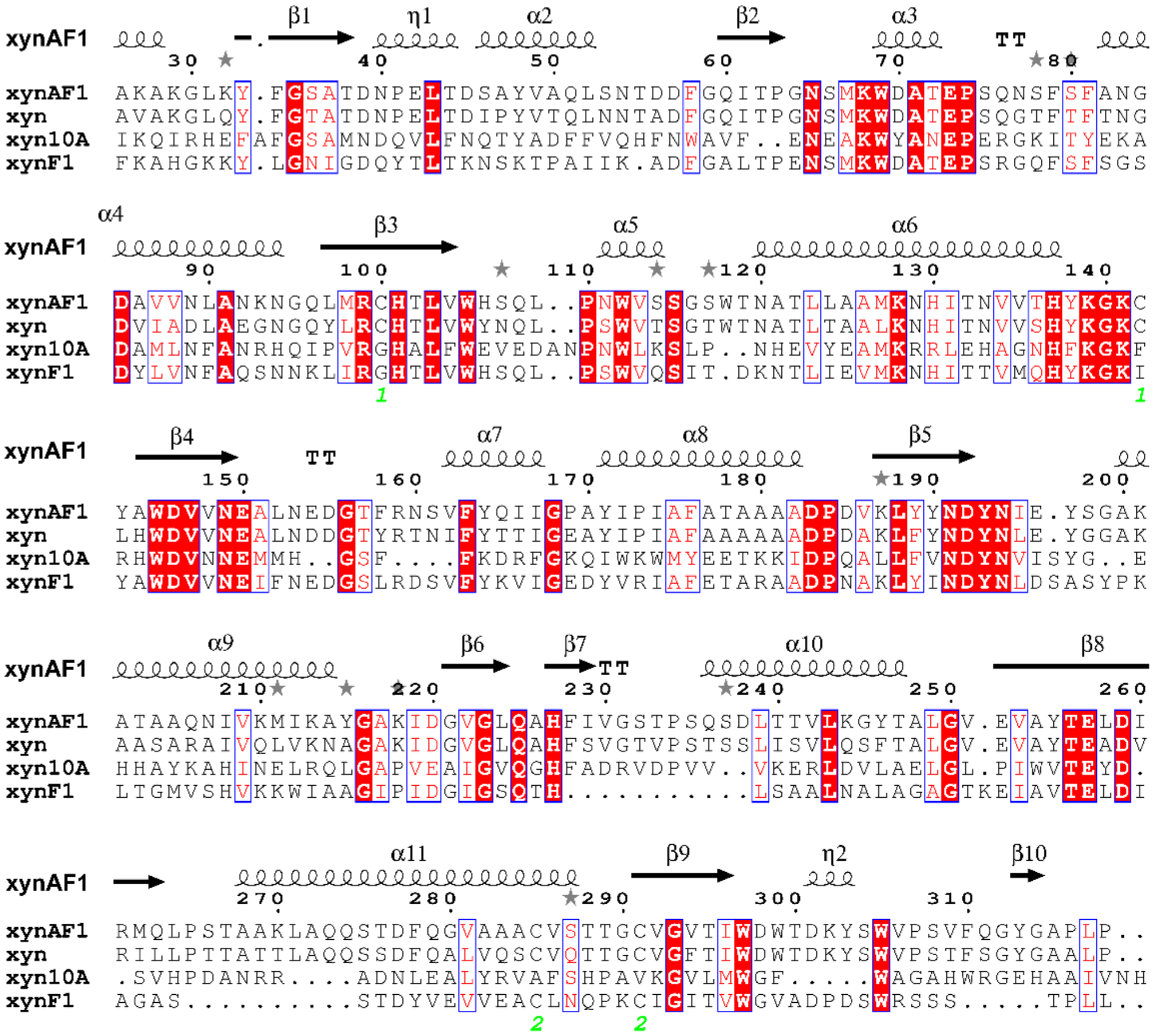

Fig. S4 Sequence alignment of XYNF1 with GH10 family xylanases. The alignment includes xynAF1 from *Aspergillus fumigatus* Z5 (NCBI ID: KMK63651.1), xyn from *Aspergillus aculeatus* ATCC 16872 (NCBI ID: OJJ95123.1), xyn10A from *Bacillus* sp. (NCBI ID: QCO69162.1), and the xynF1 from *A. niger* CBS513.88 in this study (NCBI ID: CAK38067.1). The alignment is numbered according to xynAF1. Fully conserved residues appear in white on a red background, whereas less-conserved residues appear as red letters.

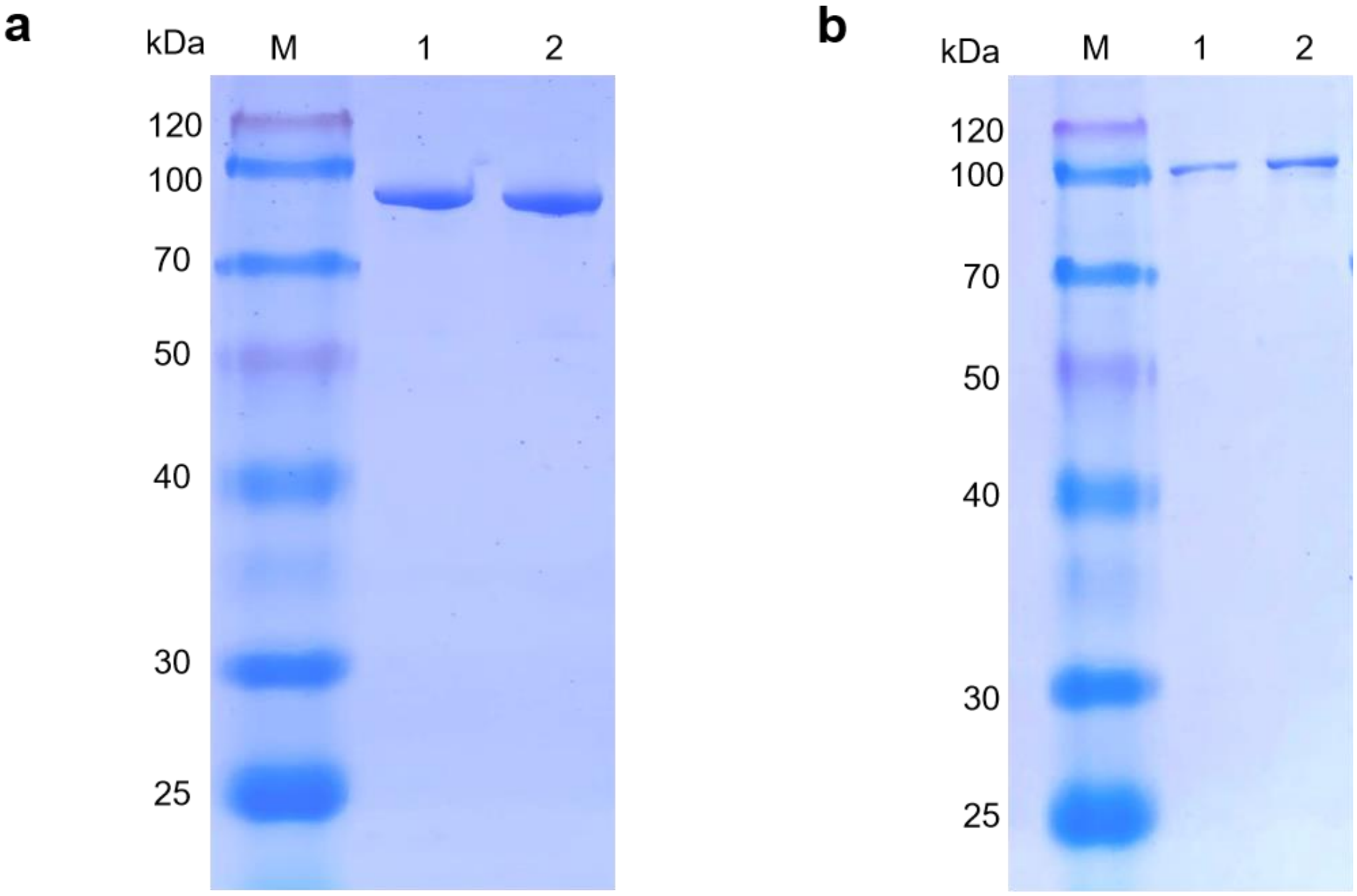

Fig. S5 SDS-PAGE analysis of purified XYNF1, XYNF1-AS, EGLD and EGLD-AS. **a** Purified XYNF1 and XYNF1-AS. 1: pCold-TF-XYNF1 protein, 2: pCold-TF-XYNF1-AS protein. **b** Purified EGLD and EGLD-AS. 1: pCold-TF-EGLD protein, 2: pCold-TF-EGLD-AS protein.
