# Supplementary material for: Alternative splicing analysis of lignocellulose-degrading enzyme genes and enzyme variants in Aspergillus niger
Source: Appl Microbiol Biotechnol. 2024 Apr 19;108(1):302. doi: 10.1007/s00253-024-13137-y (PMC11031446; doi:10.1007/s00253-024-13137-y)
Supplement: Supplementary file 1 — Supplementary file1 (PDF 716 KB) [file 253_2024_13137_MOESM1_ESM.pdf]

Alternative splicing analysis of lignocellulose-degrading enzyme genes and enzyme variants in *Aspergillus niger*

Yifan Xu, Feiyu Dong, Ruoxin Wang, Maria Ajmal, Xinyu Liu, Hui Lin* & Hongge Chen*

College of Life Sciences, Henan Agricultural University, Zhengzhou 450002, China

*Corresponding authors

Email: huilin@henau.edu.cn

Email: honggeyz@henau.edu.cn

ORCID ID: 0000-0002-7519-7341

Table S1 RT-PCR primers and intron-specific amplification primers for lignocellulose-degrading enzyme genes in *Aspergillus niger*.

Gene name	NCBI ID	RT-PCR primer sequence (5'-3')	
		Forward	Reverse
xynF1	4980082	ATGGTTCAGATCAAGGTAGCTGCA	CTAGAGAGCATTTGCGATAGCAGTG
abnC	4979546	ATGCTTTCGTTTGTCTTGCTTCT	TCACGCCACAACAGGCCA
cbhC	4982491	ATGTTTAGCCGGATTTACTTTGTCACT	TCAAAGGGAAGGATTGGCGT
bglM	4984238	ATGCACAGCATTAGTGCGCT	TTAGATAGAAAATTCCCCCGTCA
eglD	4988091	ATGAAGACTACCACCTACAGTT	TTACTGAGACGCAACGCA
		Intron spanning primer sequence (5'-3')	
xynF1	4980082	ATGAAATCTTCAACGAAGACGG	CCAGAAATTCCAGCACCTCCAC
abnC	4979546	GCACTGCCTAACGGATCCATC	CGGCTGCCCAGGGTAGAGCTA
cbhC	4982491	ATGTTTAGCCGGATTTACTTTGTCACT	GAGAAACGAGGGGCCAGATGG
		GCTGTCTAGTGCTGGGTTTGA	CCTTGACATTGCACCAGTCAC
bglM	4984238	GCCTTTGTCGCCCAGTTGA	CTGGCACCGACGTGAATACC
eglD	4988091	GACCCCGGTATCCTCTTCGAT	CAACCTTGGACTTGGACTTGG

Table S2 Lignocellulose-degrading enzyme genes in the *A. niger* CBS513.88 genome.

Number	NCBI ID	Gene name	Gene annotation	Family	DEG or no DEG
1	4977095	*xg74*	Xyloglucanase	GH74	yes
2	4977345	*cbhB*	1,4-β-D-glucan cellobiohydrolase B	GH7	yes
3	4982202	*cbhB*	1,4-β-D-glucan cellobiohydrolase B	GH7	yes
4	4987438	*aguA*	α-Glucuronidase A	GH67	yes
5	4982491	*cbhC*	1,4-β-D-glucan cellobiohydrolase C	GH6	yes
6	4985573	*cbhC*	1,4-β-D-glucan cellobiohydrolase C	GH6	yes
7	4987831	*abfB*	α-N-arabinofuranosidase B	GH54	yes
8	4978177	*abfA*	α-N-arabinofuranosidase A	GH51	no
9	4982486	*abfC*	α-N-arabinofuranosidase C	GH51	yes
10	4983621	*abfA*	α-N-arabinofuranosidase A	GH51	no

Number	NCBI ID	Gene name	Gene annotation	Family	DEG or no DEG
11	4977614	*eglA*	Endo-β-1,4-glucanase A	GH5	yes
12	4982163	*eglB*	Endo-β-1,4-glucanase B	GH5	yes
13	4989072	*eglA*	Endoglucanase A	GH5	no
14	4979546	*abnC*	α-L-arabinosidase C	GH43	yes
15	4980522	*bglJ*	β-Glucosidase J	GH3	yes
16	4981203	*bglB*	β-Glucosidase B	GH3	no
17	4982032	*bglG*	β-Glucosidase G	GH3	yes
18	4982244	*bglD*	β-Glucosidase D	GH3	yes
19	4983142	*bglD*	β-Glucosidase D	GH3	no
20	4984238	*bglM*	β-Glucosidase M	GH3	yes
21	4987033	*bglM*	β-Glucosidase M	GH3	no
22	4988081	*bglG*	β-Glucosidase G	GH3	yes
23	4989339	*bgl*	β-Glucosidase	GH3	yes
24	4989375	*bglJ*	β-Glucosidase J	GH3	no
25	4989921	*bglA*	β-Glucosidase A	GH3	yes
26	4977682	*xlnD*	Exo-1,4-β-xylosidase D	GH3	yes
27	4977958	*xynA*	Endo-1,4-β-xylanase A	GH11	yes
28	4978152	*xynA*	Endo-1,4-β-xylanase A	GH11	yes
29	4987596	*xyn5*	Endo-1,4-β-xylanase 5	GH11	yes
30	4988056	*xynB*	Endo-1,4-β-xylanase B	GH11	yes
31	4980082	*xynF1*	Endo-1,4-β-xylanase F1	GH10	yes
32	4980363	*bgl1B*	β-Glucosidase G	GH1	yes
33	4983612	-	Acetyl xylan esterase	CE5	no
34	4983631	-	Acetyl xylan esterase	CE5	yes
35	4985851	*axeA*	Acetyl xylan esterase A	CE1	yes
36	4980408	-	Hypothetical protein	CBM63	yes
37	4978139	-	Hypothetical protein	CBM50	no
38	4988766	-	Hypothetical protein	CBM48	yes
39	4979015	-	Hypothetical protein	CBM21	yes
40	4988091	*eglD*	Probable endo-β-1,4-glucanase D	CBM1/AA9	yes
41	4987123	*gun4*	Endoglucanase-4	CBM1/AA9	yes
42	4988058	-	Hypothetical protein	CBM1	yes
43	4982839	*gun4*	Endoglucanase-4	AA9	no

Number	NCBI ID	Gene name	Gene annotation	Family	DEG or no DEG
44	4985809	*gun4*	Endoglucanase-4	AA9	yes
45	4991259	*gun4*	Endoglucanase-4	AA9	no
46	4979417	*cdh*	Cellobiose dehydrogenase	AA2	yes
47	4990292	*cdh*	Cellobiose dehydrogenase	AA2	yes
48	4978017	*abr2*	Laccase abr2	AA1	yes
49	4977636	*abr2*	Laccase abr2	AA1	yes
50	4980364	*abr2*	Laccase abr2	AA1	no
51	4980991	*abr2*	Laccase abr2	AA1	yes
52	4991441	*abr2*	Laccase abr2	AA1	no
53	4985931	*lac1*	Laccase ARB	AA1	yes
54	4988595	*lac1*	Laccase ARB	AA1	yes
55	4980971	*lac2*	Laccase-2	AA1	no
56	4984589	*lac2*	Laccase-2	AA1	no

Note: DEG stands for differentially expressed genes.

Table S3 DNA sequencing verification of AS events in five selected lignocellulose-degrading enzyme genes

Gene name	Number of normal splicing transcripts in 10 transformants	Number of AS transcripts in 10 transformants
xynF1	4	6
abnC	8	2
cbhC	7	3
bglM	6	4
eglD	2	8

Note: RT-PCR products of each gene were randomly cloned and 10 transformants were selected for DNA sequencing.

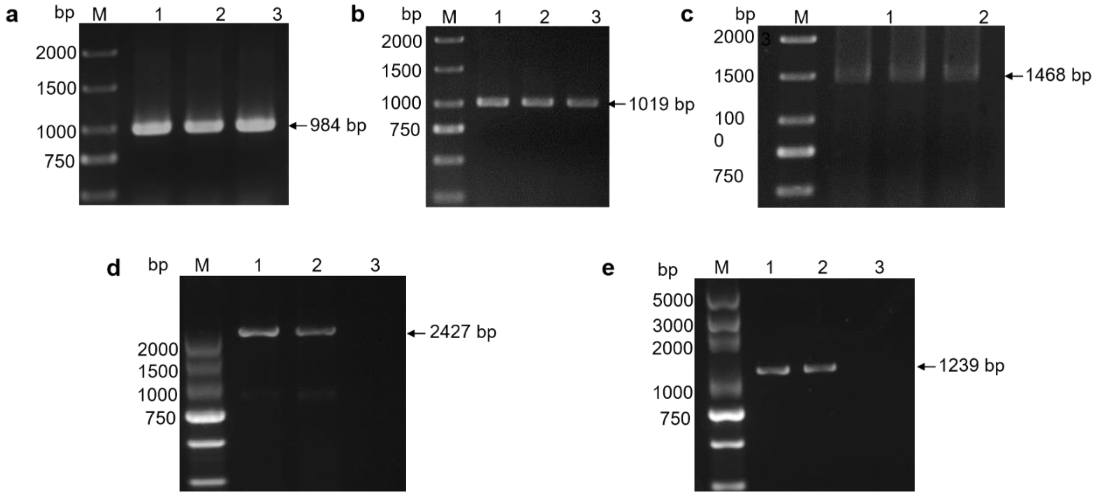

Fig. S1 RT-PCR amplification of the selected lignocellulose-degrading enzyme genes. The theoretical size of the amplified genes were marked. M: DL2000 Marker (Zhuangmeng Technology Co., Ltd., Beijing, China); 1, 2, 3: three samples in the WS group. **a** The RT-PCR electrophoresis image of the *xynF1* gene. **b** The RT-PCR electrophoresis image of the *abnC* gene. **c** The RT-PCR electrophoresis image of the *cbhC* gene. **d** The RT-PCR electrophoresis image of the *bglM* gene. **e** The RT-PCR electrophoresis image of the *eglD* gene.

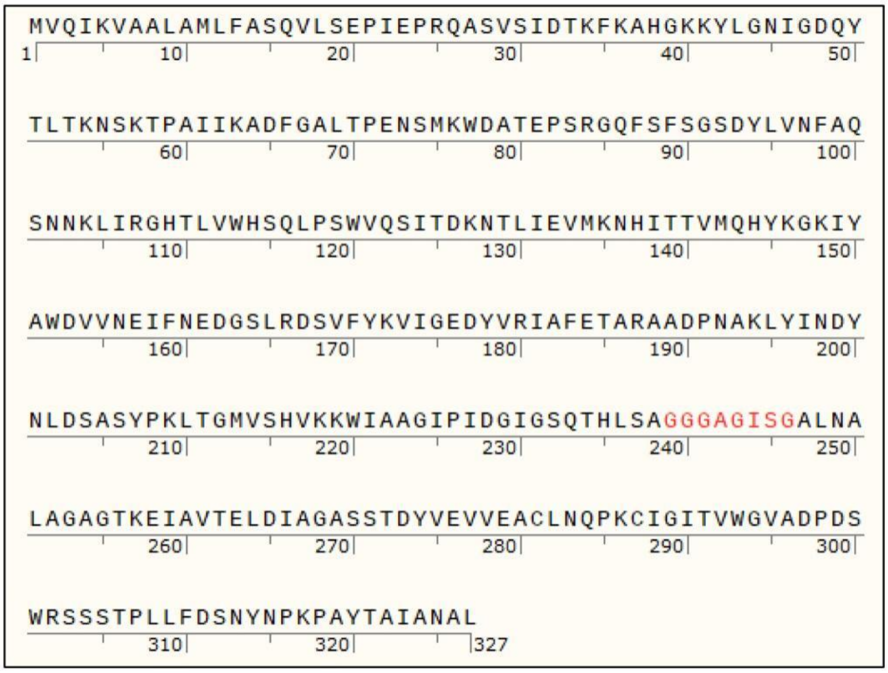

Fig. S2 Amino acid sequence of the AS variant XYNF1-AS. Amino acid changes caused by AS events are colored in red.

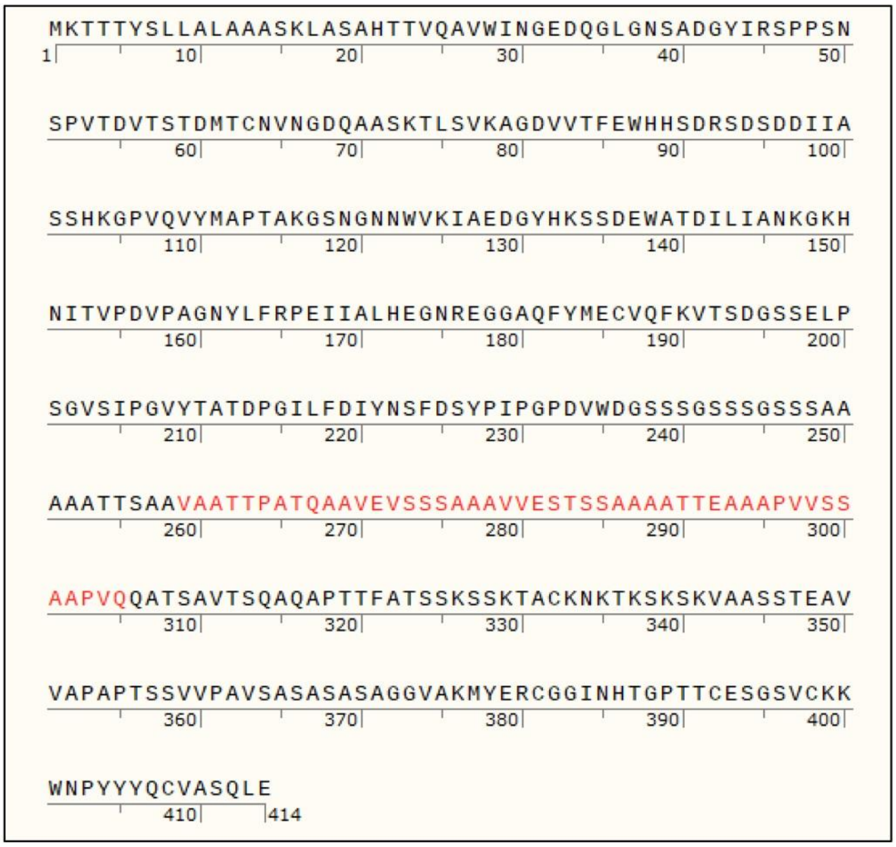

Fig. S3 Amino acid sequence of the AS variant EGLD-AS. Amino acid changes caused by AS events are colored in red.

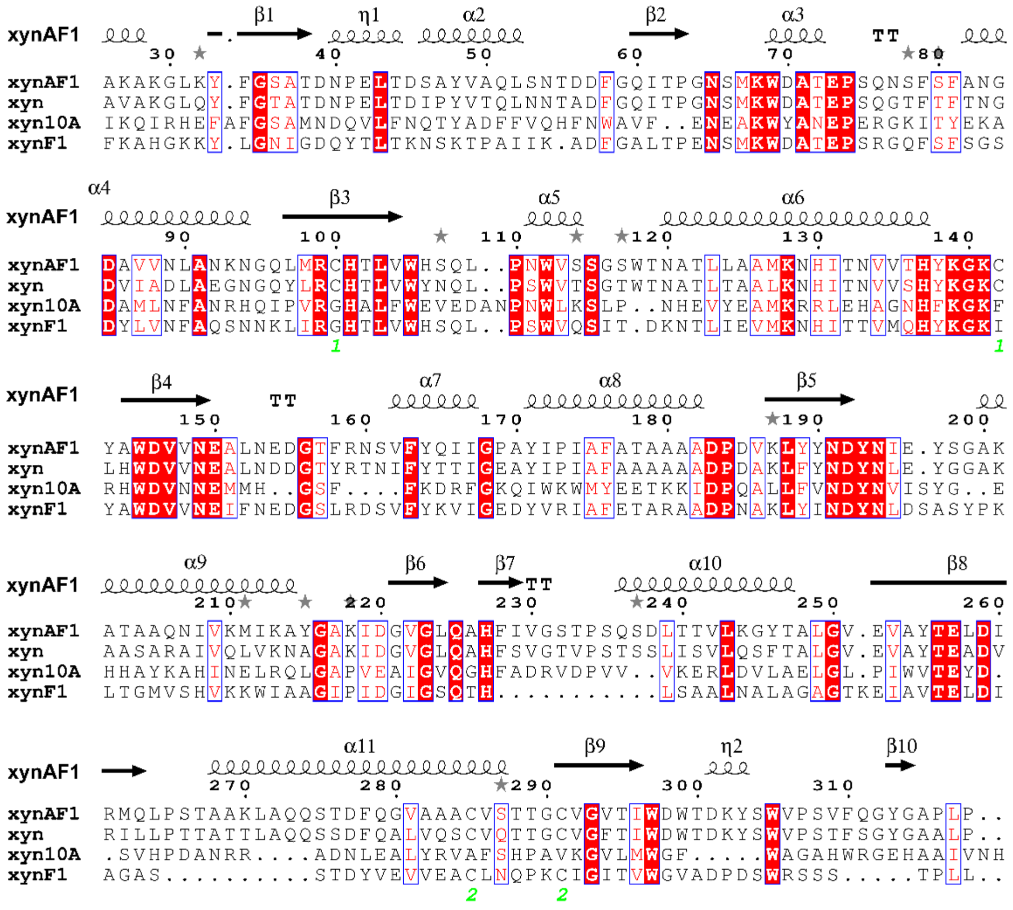

Fig. S4 Sequence alignment of XYNF1 with GH10 family xylanases. The alignment includes xynAF1 from *Aspergillus fumigatus* Z5 (NCBI ID: KMK63651.1), xyn from *Aspergillus aculeatus* ATCC 16872 (NCBI ID: OJJ95123.1), xyn10A from *Bacillus* sp. (NCBI ID: QCO69162.1), and the xynF1 from *A. niger* CBS513.88 in this study (NCBI ID: CAK38067.1). The alignment is numbered according to xynAF1. Fully conserved residues appear in white on a red background, whereas less-conserved residues appear as red letters.

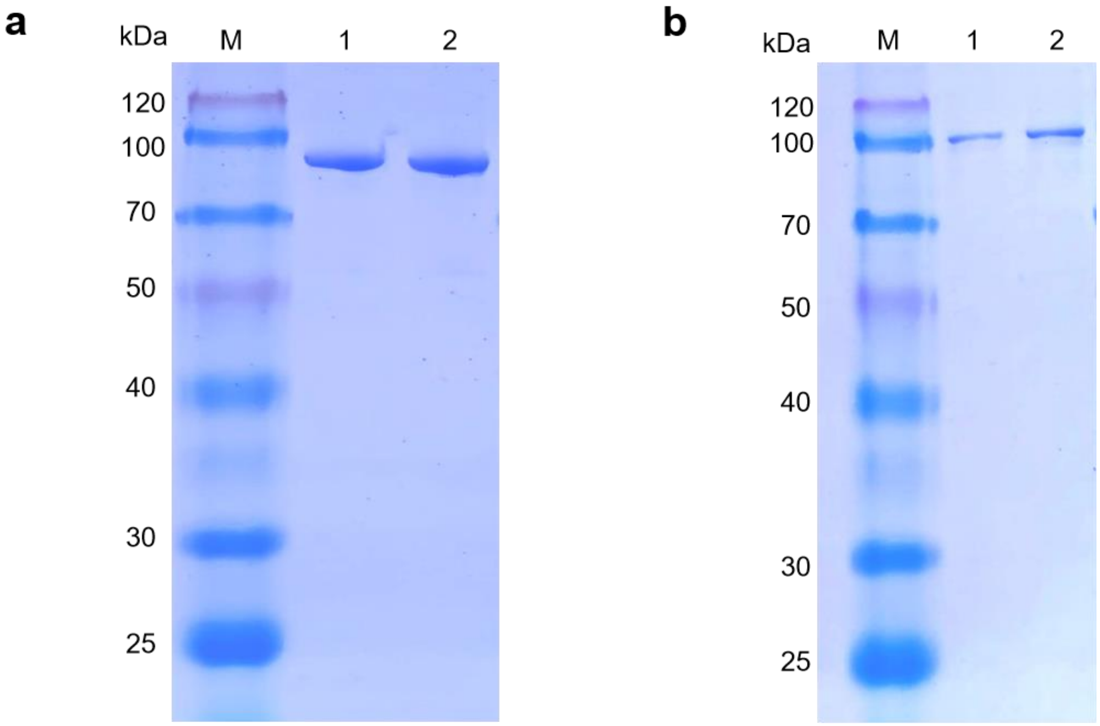

Fig. S5 SDS-PAGE analysis of purified XYNF1, XYNF1-AS, EGLD and EGLD-AS. **a** Purified XYNF1 and XYNF1-AS. 1: pCold-TF-XYNF1 protein, 2: pCold-TF-XYNF1-AS protein. **b** Purified EGLD and EGLD-AS. 1: pCold-TF-EGLD protein, 2: pCold-TF-EGLD-AS protein.
